# Development and Validation of a Machine Learning Model to Predict Anti-Drug Antibody Formation During Infliximab Induction in Crohn’s Disease

**DOI:** 10.3390/biomedicines13102464

**Published:** 2025-10-10

**Authors:** Yiting Wang, Jialin Song, Zhuoling Zheng, Xiang Peng, Xiaoyan Li, Wenjiao Wu

**Affiliations:** 1 Department of Pharmacy, The Sixth Affiliated Hospital, Sun Yat-sen University, Guangzhou 510655, China; wangyt65@mail.sysu.edu.cn (Y.W.); zhengzhling@mail.sysu.edu.cn (Z.Z.); 2 Biomedical Innovation Center, The Sixth Affiliated Hospital, Sun Yat-sen University, Guangzhou 515000, China; pengx5@mail.sysu.edu.cn; 3 Department of Orthopedics, Guangzhou Eighth People’s Hospital, Guangzhou Medical University, Guangzhou 510440, China; sjl156024@163.com; 4 Department of Gastroenterology, The Sixth Affiliated Hospital, Sun Yat-sen University, Guangzhou 510655, China; 5 Department of Pharmacy, The Affiliated Guangdong Second Provincial General Hospital of Jinan University, Guangzhou 510317, China

**Keywords:** Crohn’s disease, infliximab, anti-drug antibodies, machine learning, predictive model

## Abstract

**Background/Objectives:** The development of anti-drug antibodies (ADA) significantly diminishes the clinical efficacy of infliximab (IFX) in Crohn’s disease (CD). This study aimed to develop and validate an interpretable machine learning (ML) framework for predicting ADA risk during IFX induction therapy using multidimensional clinical and laboratory data. **Methods:** We conducted a retrospective analysis of 606 CD patients who initiated IFX induction between January 2023 and August 2024 at the Sixth Affiliated Hospital of Sun Yat-sen University. Predictor selection was performed through univariate analysis and least absolute shrinkage and selection operator (LASSO) regression, with significant features further evaluated via multivariate logistic regression. Seven ML models were developed and evaluated mainly based on area under the curve (AUC), F1 score, and Brier score. Model interpretability was enhanced using SHapley Additive exPlanations (SHAP). **Results:** Among the 606 CD patients, 145 (23.93%) developed ADA during IFX induction. Independent predictors included serum trough levels of IFX (TLI), erythrocyte sedimentation rate (ESR), history of delayed treatment, prior exposure to anti-TNF agents, and concomitant use of immunosuppressants (IMM). The XGBoost algorithm outperformed others, with an AUC of 0.899, accuracy of 0.851, F1 score of 0.640, and Brier score of 0.102 in validation. SHAP analysis identified TLI and ESR as the most influential predictors, with history of delayed treatment and prior exposure to anti-TNF agents showing moderate impact, while concomitant use of IMM was associated with a protective effect. **Conclusions:** We developed an interpretable ML model that effectively predicts ADA formation in CD patients undergoing IFX induction therapy, facilitating early risk stratification and personalized treatment planning. This approach integrates advanced analytics with clinical practice to support precision medicine in CD management.

## 1. Introduction

Crohn’s disease (CD) is a chronic and relapsing inflammatory bowel disorder with a multifactorial etiology involving genetic susceptibility, immune dysregulation, gut microbiota imbalance, and environmental factors [1]. The disease exhibits considerable clinical heterogeneity, ranging from mild symptomatic presentations to severe complications such as strictures, perforations, and fistulating disease, which substantially complicate clinical management. In patients with moderate to severe active disease, conventional therapies including corticosteroids and immunomodulators frequently fail to sustain long-term remission. Consequently, anti-tumor necrosis factor-alpha (TNF-α) biologic agents, particularly infliximab (IFX), have become integral to treatment strategies.

Despite the demonstrated efficacy of IFX, maintaining durable treatment responses remains a considerable clinical challenge. Studies report that loss of response occurs in approximately 33% of CD patients over time [2]. A pivotal mechanism contributing to treatment failure involves the formation of anti-drug antibodies (ADA), which can develop primarily through a T-cell-dependent pathway of B-cell activation [3]. These antibodies form immune complexes with IFX, promoting accelerated drug clearance, reducing serum trough levels of IFX (TLI), and potentially eliciting infusion-related reactions or hypersensitivity responses, all of which compromise therapeutic efficacy [4]. The development and persistence of ADA are influenced by numerous factors, including TLI, systemic inflammation burden, concomitant use of immunosuppressants (IMM), and genetic predisposition [5,6,7,8,9].

Previous studies have identified several predictors associated with ADA development and treatment outcomes. However, the interrelationships among these variables may involve complex nonlinear dynamics and potential effect modifications that conventional statistical methods are ill-equipped to characterize fully [10,11]. Given these limitations, machine learning (ML) presents a transformative methodological paradigm for addressing such complex clinical prediction challenges. Unlike traditional statistical approaches, ML algorithms can autonomously identify intricate nonlinear associations and higher-order interactions among features without relying on strong prior assumptions. This makes them particularly suitable for analyzing high-dimensional biomedical data and capturing subtle predictive patterns [12].

Within the field of inflammatory bowel disease (IBD) research, ML techniques are gaining increasing traction. Algorithms such as Support Vector Machine (SVM), eXtreme Gradient Boosting (XGBoost), and Light Gradient Boosting Machine (LightGBM) have been utilized to predict clinical remission and overall treatment response [13,14,15]. Nevertheless, a notable research gap persists in the development of specialized ML models focused on predicting immunogenicity to biologic agents, particularly regarding the risk of ADA formation in IFX-treated patients. Furthermore, multi-omics approaches, including genomics, microbiomics, and pharmacogenomics, hold potential for improving the prediction of ADA formation or therapeutic efficacy during IFX therapy [16,17,18,19]. These methods provide a more comprehensive understanding of the biological and environmental interactions that influence immunogenicity. For example, Sazonovs et al. demonstrated that carriage of the HLA-DQA1*05 allele markedly increases the risk of ADA formation in patients with CD [18]. Similarly, Lee et al. identified specific gut microbiome compositions, serum proteomic profiles, and metabolomic features that collectively predict an elevated risk of non-response to IFX in patients with IBD [19]. Despite these promising case studies, the integration of such multi-omics datasets into robust ML frameworks remains underdeveloped. Current research is further hampered by limitations such as restricted sample sizes, inadequate multi-dimensional data integration, suboptimal feature engineering, and poor model interpretability, which collectively hinder clinical translation and practical application [20,21,22]. 

To address these limitations, our study integrates SHAP-based interpretability with multidimensional clinical data to develop a robust and clinically applicable prediction model. Our methodology integrates multidimensional clinical and laboratory data to facilitate a systematic assessment of immunogenicity. The specific research objectives are (1) to identify robust predictors significantly associated with ADA formation. (2) to develop and validate the predictive performance of seven ML algorithms. and (3) to apply SHapley Additive exPlanations (SHAP) for interpretable modeling and elucidating variable contributions.

## 2. Materials and Methods

### 2.1. Study Population 

This retrospective cohort study enrolled patients diagnosed with CD who received IFX therapy at the Sixth Affiliated Hospital of Sun Yat-sen University between January 2023 and August 2024. All data were anonymized in compliance with institutional privacy regulations, and the study protocol was reviewed and approved by the hospital’s Ethics Committee (No. 2025ZSLYEC-377).

Participants were enrolled according to rigorously defined inclusion and exclusion criteria to ensure the reliability of the study population. Inclusion criteria were as follows: (1) age between 18 and 75 years; (2) diagnosis of CD established according to internationally recognized standards [23,24]; (3) receipt of at least four IFX infusions, in either outpatient or inpatient settings; (4) availability of complete clinical documentation. Exclusion criteria comprised (1) pregnancy or breastfeeding; (2) an incomplete IFX induction phase (i.e., less than 14 weeks); (3) missing critical clinical data. Based on institutional policy for retrospective studies, the Institutional Review Board (IRB) granted an exemption from and waived the requirement for informed consent.

### 2.2. Data Acquisition and Processing

Patient data were systematically extracted from the electronic medical record (EMR) system and the laboratory information system, encompassing the following domains: (1) Demographics and anthropometrics: sex, height, weight, body mass index (BMI), disease duration, age at disease onset, and age at the initiation of IFX treatment. (2) Disease characteristics: the Montreal classification of CD, the Crohn’s Disease Activity Index (CDAI), the presence of perianal disease, extraintestinal manifestations (EIM), complications, and the history of intestinal surgery. The Montreal classification stratifies patients based on the age, disease location and disease behavior at diagnosis, providing a comprehensive disease phenotype assessment. (3) IFX treatment: Patients received one or more IFX products, including Remicade® (Janssen Pharmaceuticals Co., Ltd., Beerse, Belgium), a biosimilar from Hisun Biopharmaceutical Co., Ltd. (Hangzhou, China), and another biosimilar from Mabtech Pharmaceuticals Co., Ltd. (Taizhou, China). Some patients switched between different IFX formulations during therapy. (4) IFX treatment details: IFX dosage during the induction phase, TLI, titers of ADA, prior exposure to anti-TNF agents, history of delayed treatment, and concomitant use of IMM. (5) Baseline laboratory parameters: erythrocyte sedimentation rate (ESR), C-reactive protein (CRP), white blood cell count (WBC), absolute neutrophil count (ANC), absolute lymphocyte count (ALC), red blood cell count (RBC), hemoglobin (HB), platelet count (PLT), absolute monocyte count (AMC), hematocrit (HCT), alanine aminotransferase (ALT), aspartate aminotransferase (AST), gamma-glutamyl transferase (GGT), alkaline phosphatase (ALP), total bilirubin (TBIL), direct bilirubin (DBIL), indirect bilirubin (IBIL), total cholesterol (TC), potassium (K), sodium (Na), calcium (Ca), phosphorus (P), uric acid (UA), creatinine (Cr), and albumin (ALB).

### 2.3. Outcome Definition of ADA Status in CD Patients

The standardized induction therapy with IFX consisted of intravenous administration at a dose of 5 mg/kg at weeks 0, 2, and 6, followed by maintenance dosing every 8 weeks. Blood samples were obtained prior to the fourth infusion (week 14). TLI and ADA titers were quantified using a commercial enzyme-linked immunosorbent assay (ELISA; Immundiagnostik, Bensheim, Germany). In accordance with established criteria [7], patients were classified as ADA-positive if their ADA titer was ≥ 10 AU/mL and ADA-negative if it was below this threshold.

### 2.4. Feature Selection

During data preprocessing, patient records with any missing values were excluded to ensure data quality and consistency. The resulting dataset was randomly divided into training and validation subsets in an 8:2 ratio. The training set served to construct a predictive model for ADA positivity, whereas the validation set was employed to evaluate its internal performance. To mitigate potential multicollinearity among predictor variables and to enhance model interpretability, a structured two-stage feature selection strategy was employed. Specifically, in the training dataset, variables showing univariate significance (*p* < 0.05) were retained as candidates for subsequent modeling. Feature selection was achieved by implementing the least absolute shrinkage and selection operator (LASSO) approach, where the lambda.1 standard error (λ.1se) criterion was applied to achieve a parsimonious model while maintaining predictive accuracy. Predictors that retained non-zero coefficients under this constraint were thereafter integrated into a multivariable logistic regression analysis, through which an optimized subset of independent variables was obtained to construct the final prediction model.

### 2.5. Model Development and Validation

Seven ML algorithms were fitted to the training dataset to construct reliable predictive models: Logistic Regression (LR), Random Forest (RF), K-Nearest Neighbors (KNN), Classification and Regression Tree (CART), SVM, XGBoost, and LightGBM. To enhance predictive accuracy, hyperparameters tuning was performed using a systematic grid search coupled with tenfold cross-validation. Subsequently, the reproducibility and effectiveness of each model were evaluated on an independent validation dataset. Additionally, the predictive performance of all models was assessed with a suite of evaluation metrics, including the area under the receiver operating characteristic curve (AUC), accuracy, sensitivity, specificity, positive predictive value (PPV), negative predictive value (NPV), F1 score, and Brier score. The AUC evaluates the model’s discriminatory power, with values between 0.5 and 0.7 indicating poor performance, 0.7 to 0.9 reflecting acceptable discrimination, and values above 0.9 demonstrating strong classification ability. The F1 score, bounded between 0 and 1, is defined as the harmonic mean of precision and recall and offers a balanced measure of classification performance, particularly in the presence of imbalanced class distributions. The Brier score, calculated as the mean squared deviation between predicted probabilities and observed outcomes, serves as an indicator of model calibration, with lower values approaching zero denoting higher predictive reliability. Final model selection was guided by an integrated evaluation of AUC, F1 score, and Brier score, focusing on an optimal balance between discriminatory power and calibration accuracy. To further assess the predictive performance of the model, receiver operating characteristic (ROC) curves, calibration plots, and decision curve analysis (DCA) were employed. ROC curves illustrate classification ability under different thresholds, with the AUC reflecting discriminative accuracy. Calibration plots assess the concordance between predicted probabilities and observed outcomes, where alignment with the 45-degree line indicates optimal calibration. Decision curve analysis quantifies the clinical utility by estimating the net benefit at varying decision thresholds, thereby supporting the model’s practical applicability.

### 2.6. Model Explainability

To enhance model interpretability, the SHAP framework was utilized to assess the impact of individual features at both the global (population-level) and local (individual-level) scales, enabling a more detailed understanding of the model’s decision logic.

### 2.7. Statistical Analysis

All statistical analyses were performed with SPSS (version 27.0, IBM Corp., Armonk, NY, USA) and R (version 4.5.0, R Foundation for Statistical Computing, Vienna, Austria). Normally distributed variables were expressed as mean ± standard deviation (Mean ± SD), and group comparisons were performed using independent sample *t*-test. Non-normally distributed variables were summarized as median and interquartile range [M (Q1, Q3)]. Categorical variables were expressed as frequencies and percentages, and comparisons were performed using the chi-square (χ^2^) test or Fisher’s exact test, depending on distribution characteristics and sample size. A two-sided *p*-value < 0.05 was considered statistically significant. The selection of statistical approaches was tailored to align with the data’s type and distribution, ensuring methodological appropriateness and strengthening the validity of the findings.

## 3. Results

### 3.1. Description of Variables

A total of 606 patients were included in this study, of whom 145 (23.93%, 145/606) tested positive for ADA during the IFX induction phase. In the training set, 116 patients were ADA-positive (23.92%, 116/485), while 29 patients in the validation set were ADA-positive (23.97%, 29/121) (Figure S1). Baseline clinical variables were compared between the training (*n* = 485) and validation (*n* = 121) sets, with no significant differences observed across any parameters (*p* > 0.05), confirming the comparability of the two sets (Table S1). Within the training set, ADA-positive patients differed significantly from ADA-negative patients in weight, BMI, TLI, ESR, Ca, ALB, history of delayed treatment, prior exposure to anti-TNF agents, concomitant use of IMM, and age at IFX initiation (all *p* < 0.05) (Table 1 and Table S2).

### 3.2. Variable Selection

**Figure 1:**
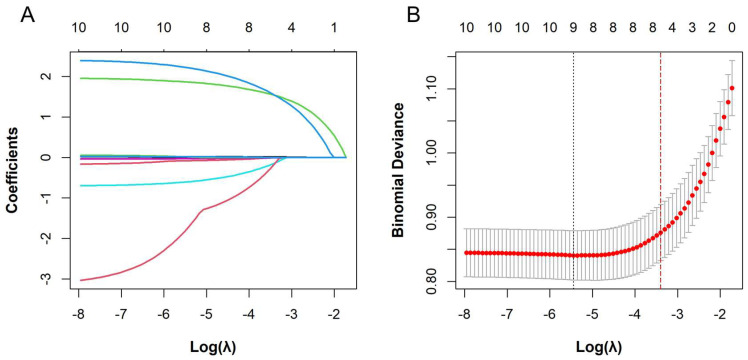
The least absolute shrinkage and selection operator (LASSO) regression analysis of anti-drug antibody positivity during infliximab induction therapy. (**A**) Variable selection path diagram derived from LASSO regression. The x-axis represents the log of the regularization parameter (log λ), and the y-axis represents the magnitude of the model coefficients. Each curve corresponds to a predictor variable: history of delayed treatment (green), prior exposure to anti-TNF agents (blue), Ca (red), use of IMM (light blue), TLI (pink), ESR (rose red), and BMI (light green). (**B**) Cross-validation curve from LASSO regression analysis. The x-axis represents log λ, and the y-axis represents binomial deviance. The numbers above the plot indicate the number of variables included at each λ value. As λ increases, fewer variables are retained in the model. The left and right dashed lines denote λ.min and λ.1se, respectively.

To mitigate potential multicollinearity among predictors, LASSO regression was applied for feature selection, including only variables that demonstrated statistical significance (*p* < 0.05) in the univariate analysis. As the regularization parameter (λ) increased, coefficients of less informative predictors were progressively shrunk toward zero. This process identified seven predictors with non-zero coefficients: BMI, TLI, ESR, Ca, history of delayed treatment, prior exposure to anti-TNF agents, and use of IMM (Figure 1). Multivariate logistic regression further demonstrated that TLI, ESR, history of delayed treatment, prior exposure to anti-TNF agents, and concomitant use of IMM were independently associated with ADA positivity during the IFX induction phase (*p* < 0.05) (Table 2).

### 3.3. Assessment of Predictive Model Performance

**Figure 2:**
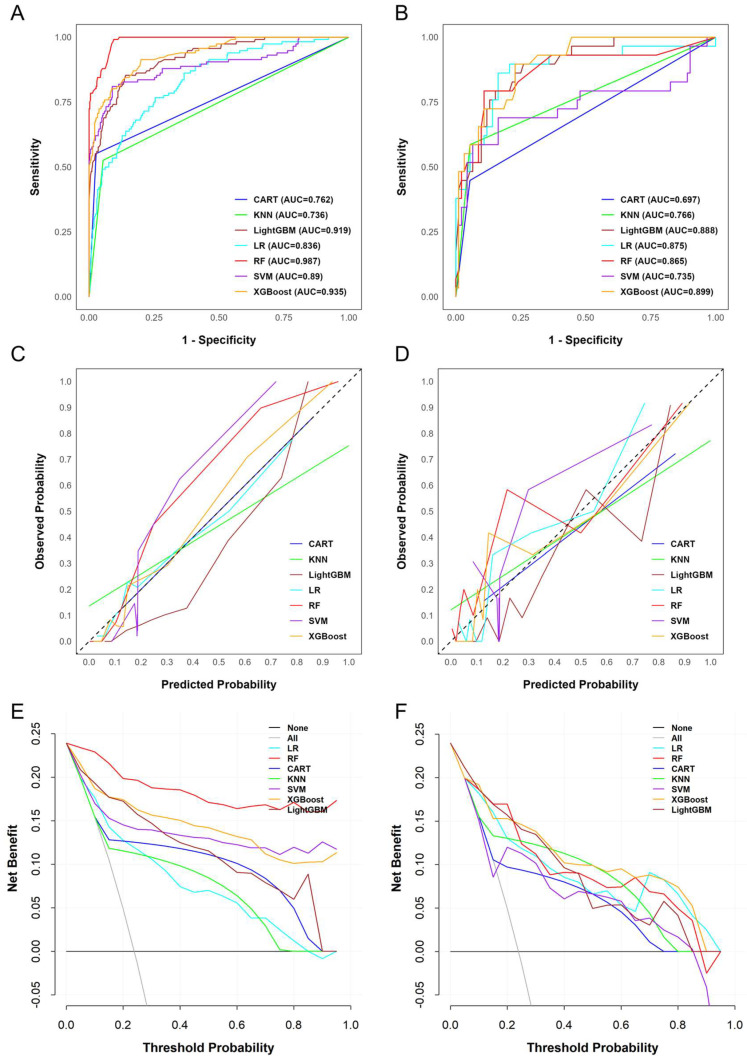
Machine learning models for predicting anti-drug antibody formation during infliximab induction. (**A**) Receiver operating characteristic (ROC) curve for the training set. (**B**) ROC curve for the validation set. (**C**) Calibration plot for the training set. (**D**) Calibration plot for the validation set. (**E**) Decision curve analysis (DCA) plot for the training set. (**F**) DCA plot for the validation set.

The model’s hyperparameters were optimized using grid search with ten-fold cross-validation, and the final settings are shown in Table S3. A systematic evaluation of seven ML models was performed to examine their predictive performance in the training and validation sets (Table S4 and Figure 2). Regarding discrimination, RF achieved the highest AUC in the training set (0.987, 95% CI: 0.980–0.994), followed by XGBoost (0.935, 95% CI: 0.910–0.961) and LightGBM (0.919, 95% CI: 0.891–0.948). However, RF showed a substantial decrease in discrimination in the validation set (AUC = 0.865), whereas XGBoost maintained a high AUC of 0.899, demonstrating better generalizability. Moreover, although LightGBM also showed high discriminatory ability (AUC = 0.888), it was slightly less stable than XGBoost. By comparison, CART and SVM yielded lower AUC values, indicating weaker discriminative performance. Furthermore, an evaluation of classification balance using the F1 score further differentiated the models. Although RF achieved the highest F1 score in the training set (0.842), it declined sharply to 0.612 in the validation set, suggesting overfitting. In contrast, XGBoost preserved a more favorable balance between precision and recall, with F1 scores of 0.768 in the training set and 0.640 in the validation set, outperforming RF and the other models overall. While LightGBM produced moderate F1 scores, CART and SVM performed poorly. 

In addition, calibration analyses provided further insights. As shown in Figure 2C,D, the predicted probabilities generated by XGBoost closely matched the observed outcomes, whereas RF exhibited clear miscalibration in the validation cohort. This was also reflected in the Brier scores: RF had the lowest score in the training set (0.048) but increased to 0.117 in the validation set, highlighting its lack of robustness. In contrast, XGBoost maintained low and stable Brier scores across both datasets (0.077 in training and 0.102 in validation), indicating more reliable probability estimation. Finally, decision curve analysis (Figure 2E,F) indicated that XGBoost demonstrated superior net clinical benefit over a wide range of threshold probabilities, outperforming other models. 

### 3.4. SHAP-Based Interpretation of ADA Prediction in the XGBoost Model

To improve the interpretability of the XGBoost model, we applied SHAP analysis. This method quantifies each feature’s contribution to predictions by assigning SHAP values, which represent the marginal impact relative to the average prediction. Positive SHAP values increase the predicted risk of ADA positivity, whereas negative values decrease it. In visualizations, yellow indicates positive contributions (elevating risk), and red denotes negative contributions (lowering risk).

**Figure 3:**
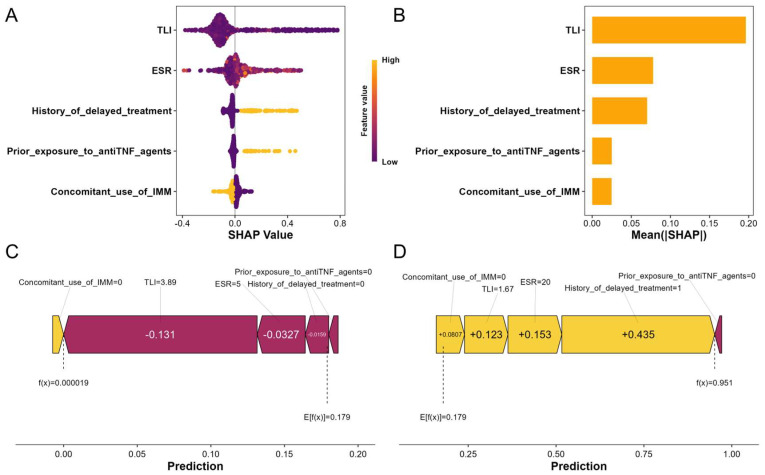
Shapley additive explanations (SHAP)-based interpretation of the extreme gradient boosting (XGBoost) model. (**A**) SHAP beeswarm plot showing the distribution of SHAP values for each feature in ADA risk prediction. Dots represent individual patients (red: higher values; blue: lower values). Positive values (right) indicate increased risk, whereas negative values (left) suggest reduced risk. (**B**) SHAP bar plot showing feature importance based on mean absolute SHAP values. (**C**) SHAP force plot for a patient who remained anti-drug antibody (ADA)-negative during infliximab (IFX) induction. (**D**) SHAP force plot for a patient who developed ADA-positivity during IFX induction. SHAP values illustrate the contribution of each feature to ADA positivity. f(X) is the predicted probability for a sample, and E[f(X)]. E[f(X)] is the baseline (mean prediction across the dataset). Yellow features (left) indicate increased risk, red features (right) indicate reduced risk, and arrow length reflects the magnitude of the effect.

At the global level, summary plots (Figure 3A,B) demonstrate that TLI exerted the greatest influence on predictions, followed by ESR and history of delayed treatment. These features primarily drove model performance. TLI and ESR exhibited wide SHAP value distributions, reflecting their significant and varied impact across patients. In contrast, prior exposure to anti-TNF agents and concomitant use of IMM showed lower mean absolute SHAP values, indicating minimal overall influence. These observations correspond closely to the outcomes of the ablation analysis (Figure S2 and Table S5).

At the individual level, SHAP force plots (Figure 3C,D) offer detailed insights into how feature values affect predictions. This enables clinicians to follow the model’s decision process for personalized risk assessment. For example, in a patient with low predicted ADA risk (Figure 3C), a TLI of 3.89 ug/mL contributed negatively (red bar, SHAP value < 0). This was the main factor reducing risk, supported by low ESR and no delayed treatment history. Such a profile suggests standard IFX dosing, without need for immediate intensification or adjunctive therapies, due to adequate drug exposure and controlled inflammation. In contrast, for a patient with high predicted risk (Figure 3D), history of delayed treatment was the primary positive contributor (yellow bar, SHAP value +0.435). An elevated ESR of 20 mm/h and suboptimal TLI of 1.67 ug/mL further increased the risk. This scenario may warrant interventions, such as proactive therapeutic drug monitoring to optimize TLI, early IMM co-therapy, or dose adjustments to reduce immunogenicity before the fourth infusion.

**Figure 4:**
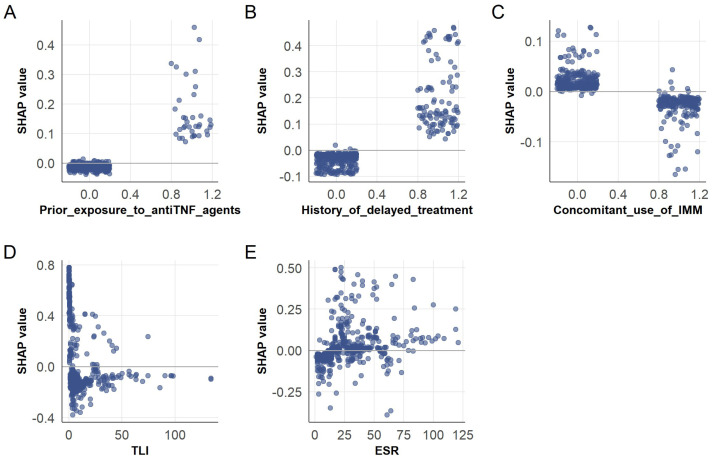
Shapley additive explanations (SHAP) dependence plots for interpreting feature influence on extreme gradient boosting (XGBoost) model predictions. (**A**) Prior exposure to anti-TNF agents; (**B**) History of delayed treatment; (**C**) Concomitant use of immunosuppressants (IMM); (**D**) Serum trough levels of infliximab (TLI); (**E**) Erythrocyte sedimentation rate (ESR). SHAP dependence plots illustrate how individual features influence model predictions, with each point representing a patient. The vertical axis shows the SHAP value, indicating the direction and magnitude of feature contribution, while the horizontal axis displays the actual feature value. A positive SHAP value reflects an increased likelihood of an ADA-positive outcome. Color gradients denote data point density, with darker regions reflecting higher patient concentrations.

SHAP dependence plots (Figure 4) illustrate feature interactions with model outputs. Colored scatter points highlight nonlinear relationships and clinical thresholds. For binary features, including prior anti-TNF exposure (Figure 4A), history of delayed treatment (Figure 4B), and concomitant use of IMM (Figure 4C), SHAP values clustered near zero, with occasional positive spikes. This confirms their limited influence but suggests targeted utility in subgroups, such as increased monitoring for patients with prior anti-TNF exposure. Continuous features showed distinct patterns. TLI (Figure 4D) spanned a broad SHAP range, with lower values markedly increasing risk and higher values conferring protection. ESR (Figure 4E) exhibited a positive trend, with higher values linked to increased risk, reinforcing its role as an inflammation marker for identifying patients needing intensified immunosuppression.

Collectively, these analyses clarify the model’s decision-making through distinct visual indicators of direction and magnitude. They offer practical recommendations, including initiating AZA co-therapy or adjusting IFX doses for patients with low TLI and elevated ESR, while maintaining standard IFX regimens for those without delayed treatment. At the patient-specific level, tailored insights involve proactive TLI monitoring and adding AZA when ESR indicates significant inflammation, aligning with evidence-based strategies to reduce ADA risk. This framework integrates interpretable artificial intelligence with clinical practice, meeting the need for precise tools and patient-centered insights to enhance CD management. 

## 4. Discussion

This real-world investigation employed a ML framework to develop a predictive model for the occurrence of ADA in patients with CD initiating IFX induction therapy. Among the algorithms tested, the XGBoost algorithm demonstrated superior and stable predictive performance, with an AUC of 0.899 in the validation set, highlighting the feasibility of early ADA risk stratification using routine clinical parameters. SHAP analysis identified TLI, ESR, concomitant use of IMM, prior exposure to anti-TNF agents, and history of delayed treatment as major predictors. The model demonstrated not only strong discriminative ability but also high interpretability, thereby laying the groundwork for clinical application.

Our findings are broadly aligned with previous studies but also yield several novel insights. First, ADA was reaffirmed as a key driver of accelerated clearance and reduced TLI, ultimately impairing therapeutic efficacy [10,25,26,27]. Notably, the incidence of ADA in the low-TLI subgroup reached 48%, substantially higher than the 28% reported in prior studies [28]. This observation supports the concept of a “TLI-ADA vicious cycle”, in which accelerated clearance leads to insufficient exposure, impaired immune tolerance, and subsequent ADA formation. Real-world factors such as irregular dosing, underutilization of IMM, and patient heterogeneity may aggravate this cycle, underscoring the importance of maintaining adequate drug levels.

Second, inflammatory activity also emerged as another important driver of ADA development. The positive association between elevated ESR and ADA risk is in line with the concept that active inflammation enhances innate immunity and antigen presentation, thereby amplifying immunogenic responses [29,30]. Our study also suggested that patients with elevated ESR may exhibit poorer nutritional status, as indicated by lower HB and ALB levels (Figure S5). These findings could provide further insight into the clinical heterogeneity of this population. Third, regarding the protective role of concomitant IMM, multiple studies have indicated that combination therapy with IMM reduces ADA incidence by modulating immune activation. Reported ADA rates range from 4%–20% with monotherapy versus 4%–6% with combination regimens [31,32,33]. Similarly, our analysis showed significantly higher ADA incidence among patients not receiving IMM. Furthermore, prior exposure to anti-TNF agents and delayed IFX administration were also identified as risk factors [10,34,35,36,37]. For instance, Ben et al. [35] showed that infusion delays >3 days reduced TLI by over 20%, and Brun et al. [10] reported that delays >3 weeks increased ADA risk fourfold (OR: 4.12, 95% CI: 1.23–13.75). Consistent with these observations, our results further revealed that treatment delay was significantly associated with elevated ESR and reduced TLI (*p* < 0.05) (Figures S3 and S4), thereby reinforcing the evidence that delayed administration can adversely affect both immunogenicity and therapeutic exposure.

Nevertheless, some findings diverged from established literature. Notably, the contribution of concomitant use of IMM to the model was less pronounced than that of TLI or ESR, contrasting with previous studies highlighting IMM as a key protective factor [38]. The SONIC trial established that maintaining higher drug concentrations is the primary factor in suppressing ADA formation, regardless of concomitant IMM use [32]. Moreover, in real-world settings, the efficacy of IMM in curtailing ADA development may also be attenuated by factors such as insufficient dosing, abbreviated treatment duration, or poor patient adherence. Similarly, CRP, although a widely used inflammatory marker in IBD [39,40], was not a significant predictor in our model. This may reflect the “CRP non-responder” phenotype observed in some patients [41], wherein CRP levels remain low despite active inflammation. As CRP production is influenced by gene polymorphisms, such variations may further compromise its reliability in certain CD patients [41,42], limiting its predictive value. Importantly, a subset of patients developed ADA despite adequate TLI and low ESR, suggesting that immunogenicity is also shaped by intrinsic immune background and genetic predisposition. Prior studies linking ADA formation to HLA-DQA1*05 [18], FCGR3A polymorphisms [43], and microbiome alterations [44] highlights the need for multi-omics integration in future models.

Model interpretability represents a key strength of this study. Unlike conventional regression models or “black-box” deep learning approaches, SHAP allows explicit quantification of feature contributions at both cohort and individual levels [45,46]. Clinicians can not only identify high-risk patients but also recognize key contributing factors such as low TLI, elevated ESR, and absence of IMM co-therapy, thereby enabling timely interventions to mitigate ADA development. Such interpretability facilitates integration into EMR for automated risk alerts and personalized treatment recommendations. Moreover, interpretable models align with ethical principles, support patient education, and encourage shared decision-making, ultimately improving adherence and outcomes. As clinical decision support systems gain prominence in chronic disease management, our model and interpretability framework provide an important technical basis for individualized IBD treatment, prevention of therapeutic failure, and rational allocation of healthcare resources.

While this study highlights strengths in its modeling approach and clinical interpretability, some limitations should be considered. First, as a retrospective, single-center study with a relatively small sample size, exclusion of patients with missing data, and lack of external validation, the results may be prone to selection bias and have limited generalizability. Additionally, our retrospective data did not routinely collect prior ADA results for anti-TNF therapies, which could impact the interpretation of immunogenicity. Second, the model primarily relies on clinical data and does not include molecular factors, such as HLA-DQA1*05 genotyping [18], FcγR polymorphisms [43], or gut microbiome composition [47]. Integrating multi-omics data, including genomics and microbiomics, could improve predictive accuracy and provide a deeper understanding of the mechanisms behind immunogenicity. Future research should incorporate multi-center, prospective cohorts to better assess the robustness and broader applicability of the model. Third, the current model offers static predictions and does not account for the timing of ADA formation or fluctuations in ADA titers. We suggest incorporating time-to-event methods, such as survival analysis to capture ADA onset, and time-series techniques, such as long short-term memory networks [48], to create dynamic risk monitoring tools. Finally, although SHAP improves interpretability, the model has not yet been integrated into EMR systems or automated intervention workflows. Future efforts should focus on optimizing user-friendly interfaces and confirming the model’s clinical value through real-world validation.

## 5. Conclusions

In conclusion, our study identified key clinical features associated with IFX immunogenicity and demonstrated the superior predictive performance of XGBoost in predicting ADA formation. These findings provide valuable insights for identifying high-risk patients and hold promise for supporting personalized management strategies for CD patients receiving IFX therapy, ultimately aiming to optimize treatment outcomes and minimize adverse events.

## Figures and Tables

**Table 1:** Comparison of other clinical characteristics between ADA-positive and ADA-negative patients during IFX induction therapy in the training set.

Variables	Total (*n* = 485)	ADA Negative (*n* = 369)	ADA Positive (*n* = 116)	*p* Value
Sex, *n* (%)				0.187
Male, *n* (%)	375 (77.32%)	291 (78.86%)	84 (72.41%)	
Female, *n* (%)	110 (22.68%)	78 (21.14%)	32 (27.59%)	
BMI (kg/m^2^), M (Q_1_, Q_3_)	19.22 (17.44, 20.90)	19.47 (17.74, 20.96)	18.46 (17.08, 20.47)	0.013
Age at onset (yr), M (Q_1_, Q_3_)	25.00 (20.00, 30.00)	25.00 (20.00, 30.00)	25.00 (21.00, 30.00)	0.409
Age at initiation of IFX (yr), M (Q_1_, Q_3_)	28.00 (23.00, 34.00)	28.00 (23.00, 33.00)	29.50 (24.00, 36.00)	0.044
Disease duration (yr), M (Q_1_, Q_3_)	2.00 (1.00, 5.00)	2.00 (1.00, 5.00)	2.00 (1.00, 7.00)	0.148
Age at diagnosis, *n* (%)				0.336
<16	29 (5.98%)	24 (6.50%)	5 (4.31%)	
16–40	426 (87.84%)	325 (88.08%)	101 (87.07%)	
>40	30 (6.19%)	20 (5.42%)	10 (8.62%)	
Location at diagnosis, *n* (%)				0.249
L1	53 (10.93%)	46 (12.47%)	7 (6.03%)	
L2	20 (4.12%)	16 (4.34%)	4 (3.45%)	
L3	375 (77.32%)	280 (75.88%)	95 (81.90%)	
L4	37 (7.63%)	27 (7.32%)	10 (8.62%)	
Behavior at diagnosis, *n* (%)				0.860
B1	275 (56.70%)	210 (56.91%)	65 (56.03%)	
B2	80 (16.49%)	59 (15.99%)	21 (18.10%)	
B3	130 (26.80%)	100 (27.10%)	30 (25.86%)	
CDAI, n (%)				0.338
remission	100 (20.62%)	74 (20.05%)	26 (22.41%)	
mild	248 (51.13%)	193 (52.30%)	55 (47.41%)	
moderate	124 (25.57%)	90 (24.39%)	34 (29.31%)	
severe	13 (2.68%)	12 (3.25%)	1 (0.86%)	
Perianal disease, *n* (%)				0.530
No	166 (34.23%)	123 (33.33%)	43 (37.07%)	
Yes	319 (65.77%)	246 (66.67%)	73 (62.93%)	
EIM, *n* (%)				0.497
No	405 (83.51%)	311 (84.28%)	94 (81.03%)	
Yes	80 (16.49%)	58 (15.72%)	22 (18.97%)	
Complications, *n* (%)				1.000
No	242 (49.90%)	184 (49.86%)	58 (50.00%)	
Yes	243 (50.10%)	185 (50.14%)	58 (50.00%)	
History of intestinal surgery, *n* (%)				0.201
No	343 (70.72%)	255 (69.11%)	88 (75.86%)	
Yes	142 (29.28%)	114 (30.89%)	28 (24.14%)	
History of delayed treatment, *n* (%)				<0.001
No	382 (78.76%)	326 (88.35%)	56 (48.28%)	
Yes	103 (21.24%)	43 (11.65%)	60 (51.72%)	
Prior exposure to anti-TNF agents, *n* (%)				<0.001
No	449 (92.58%)	359 (97.29%)	90 (77.59%)	
Yes	36 (7.42%)	10 (2.71%)	26 (22.41%)	
Concomitant use of IMM, *n* (%)				0.039
No	284 (58.56%)	206 (55.83%)	78 (67.24%)	
Yes	201 (41.44%)	163 (44.17%)	38 (32.76%)	
Dosage (mg/kg), M (Q_1_, Q_3_)	5.71 (5.17, 6.25)	5.66 (5.17, 6.12)	5.88 (5.19, 6.38)	0.053
TLI (ug/mL), M (Q_1_, Q_3_)	4.68 (2.02, 11.72)	6.20 (3.23, 13.66)	0.79 (0.40, 3.97)	<0.001
ESR (mm/h), M (Q_1_, Q_3_)	22.00 (11.00, 38.00)	19.00 (8.00, 35.00)	28.50 (20.00, 47.50)	<0.001
Ca (mmol/L), M (Q_1_, Q_3_)	2.29 (2.21, 2.38)	2.30 (2.22, 2.39)	2.25 (2.19, 2.33)	0.003
ALB (g/L), Mean ± SD	39.83 ± 5.10	40.23 ± 5.02	38.57 ± 5.14	0.003

BMI—body mass index; IFX—Infliximab; ADA—anti-drug antibodies; CDAI—the Crohn’s disease activity index; L1—ileal; L2—colonic; L3—ileocolonic location of disease; L4—upper gastrointestinal; B1—inflammatory disease; B2—stricturing disease; B3—penetrating disease; EIM—extraintestinal manifestations; IMM—immunosuppressants; TLI—serum trough levels of IFX; ESR—erythrocyte sedimentation rate; CRP—C-reactive protein; Ca—calcium; ALB—albumin.

**Table 2:** Multivariable logistic regression analysis of factors associated with ADA positivity during IFX induction therapy.

Variables	β	SE	Wald χ^2^	OR (95% CI)	*p* Value
Prior exposure to anti-TNF agents	2.406	0.457	27.682	11.091 (4.673, 28.415)	<0.001
History of delayed treatment	1.935	0.277	48.943	6.926 (4.049, 12.005)	<0.001
Concomitant use of IMM	−0.709	0.275	6.649	0.492 (0.283, 0.836)	0.010
TLI	−0.037	0.013	8.184	0.964 (0.937, 0.986)	0.004
ESR	0.019	0.006	8.814	1.019 (1.008, 1.030)	<0.001
BMI	−0.09	0.048	3.470	0.914 (0.830, 1.003)	0.062
Ca	−1.779	0.978	3.311	0.169 (0.024, 1.112)	0.069

ADA—anti-drug antibodies; IFX—Infliximab; IMM—immunosuppressants; TLI—serum trough levels of IFX; ESR—erythrocyte sedimentation rate; BMI—body mass index; Ca—calcium; SE—standard error.

## Data Availability

The datasets used and analyzed in this study are available from the corresponding author upon reasonable request. Access to the data is restricted due to privacy concerns.

## Supplementary Materials

The following supporting information can be downloaded at https://www.mdpi.com/article/10.3390/biomedicines13102464/s1. Figure S1: Flow chart for patient selection; Figure S2: Key predictors of the XGBoost model identified through ablation study; Figure S3: Comparison of CDAI score, age at onset, BMI, and complication frequencies between the delayed-treatment group and the non-delayed-treatment group; Figure S4: Comparisons of erythrocyte sedimentation rate (ESR), hemoglobin (HB), albumin (ALB), and serum trough levels of infliximab (TLI) between the delayed-treatment group and the non-delayed-treatment group; Figure S5: Comparison of hemoglobin (HB), age at onset, BMI, albumin (ALB), and serum trough levels of infliximab (TLI) stratified by erythrocyte sedimentation rate (ESR) level (>15 mm/h vs. ≤15 mm/h); Table S1: Comparison of characteristics between the training and validation cohorts; Table S2: Comparison of other clinical characteristics between ADA-positive and ADA-negative patients during IFX induction therapy in the training set; Table S3: Hyperparameter configurations for machine learning models; Table S4: Model performance in predicting ADA positivity during IFX induction therapy in the training and validation sets; Table S5: Ablation study of the XGBoost model: performance evaluation on training and testing sets.
