# Supplementary material for: Development and Validation of a Machine Learning Model to Predict Anti-Drug Antibody Formation During Infliximab Induction in Crohn’s Disease
Source: Biomedicines. 2025 Oct 10;13(10):2464. doi: 10.3390/biomedicines13102464 (PMC12561279; doi:10.3390/biomedicines13102464)
Supplement: Supplementary file 1 [file biomedicines-13-02464-s001.zip › biomedicines-3876321-supplementary.pdf]

Supplementary materials

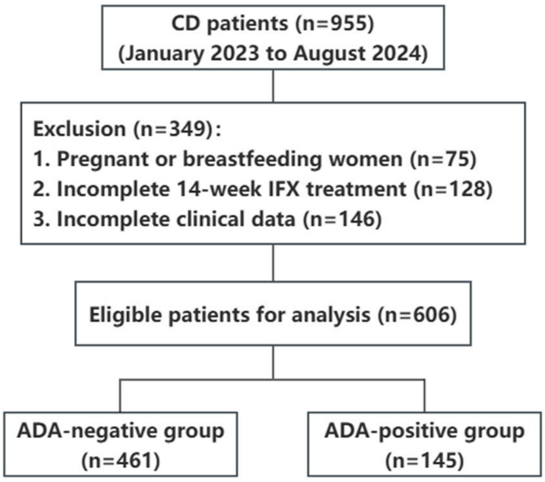

Figure S1. Flow chart for patient selection

Table S1. Comparison of characteristics between the training and validation cohorts

Variables	Total (*n*=606)	Training set (*n*=485)	Validation set (*n*=121)	*p* value
Sex, n (%)				0.081
Male, *n* (%)	459 (75.74%)	375 (77.32%)	84 (69.42%)	
Female, *n* (%)	147 (24.26%)	110 (22.68%)	37 (30.58%)	
Height (cm), M (Q1, Q3)	169.00 (163.00, 173.00)	169.00 (163.00, 173.00)	168.00 (160.00, 173.00)	0.381
Weight (kg), M (Q1, Q3)	54.00 (48.00, 61.00)	54.00 (48.00, 61.00)	53.00 (49.00, 62.00)	0.791
BMI (kg/m^2), M (Q1, Q3)	19.19 (17.51, 20.98)	19.22 (17.44, 20.90)	19.16 (17.59, 21.45)	0.615
Age at onset (year), M (Q1, Q3)	25.00 (20.00, 30.00)	25.00 (20.00, 30.00)	24.00 (19.00, 31.00)	0.415
Age at initiation of IFX (year), M (Q1, Q3)	28.00 (23.00, 34.00)	28.00 (23.00, 34.00)	27.00 (22.00, 35.00)	0.836
Disease duration(yr), M (Q1, Q3)	2.00 (1.00, 5.00)	2.00 (1.00, 5.00)	2.00 (1.00, 6.00)	0.274
Age at diagnosis, *n* (%)				0.393
<16	40 (6.60%)	29 (5.98%)	11 (9.09%)	
16-40	527 (86.96%)	426 (87.84%)	101 (83.47%)	
>40	39 (6.44%)	30 (6.19%)	9 (7.44%)	
Location at diagnosis, *n* (%)				0.411
L1	64 (10.56%)	53 (10.93%)	11 (9.09%)	
L2	27 (4.46%)	20 (4.12%)	7 (5.79%)	
L3	464 (76.57%)	375 (77.32%)	89 (73.55%)	
L4	51 (8.42%)	37 (7.63%)	14 (11.57%)	
Behavior at diagnosis, *n* (%)				0.807
B1	347 (57.26%)	275 (56.70%)	72 (59.50%)	
B2	100 (16.50%)	80 (16.49%)	20 (16.53%)	

B3	159 (26.24%)	130 (26.80%)	29 (23.97%)	
CDAI, *n* (%)				0.512
remission	121 (19.97%)	100 (20.62%)	21 (17.36%)	
mild	312 (51.49%)	248 (51.13%)	64 (52.89%)	
moderate	154 (25.41%)	124 (25.57%)	30 (24.79%)	
severe	19 (3.14%)	13 (2.68%)	6 (4.96%)	
Perianal disease, *n* (%)				1.000
No	208 (34.32%)	166 (34.23%)	42 (34.71%)	
Yes	398 (65.68%)	319 (65.77%)	79 (65.29%)	
EIM, n (%)				1.000
No	506 (83.50%)	405 (83.51%)	101 (83.47%)	
Yes	100 (16.50%)	80 (16.49%)	20 (16.53%)	
Complications, *n* (%)				0.655
No	299 (49.34%)	242 (49.90%)	57 (47.11%)	
Yes	307 (50.66%)	243 (50.10%)	64 (52.89%)	
History of intestinal surgery, *n* (%)				0.886
No	430 (70.96%)	343 (70.72%)	87 (71.90%)	
Yes	176 (29.04%)	142 (29.28%)	34 (28.10%)	
History of delayed treatment, *n* (%)				0.305
No	483 (79.70%)	382 (78.76%)	101 (83.47%)	
Yes	123 (20.30%)	103 (21.24%)	20 (16.53%)	
Prior exposure to anti-TNF agents, *n* (%)				0.904
No	560 (92.41%)	449 (92.58%)	111 (91.74%)	
Yes	46 (7.59%)	36 (7.42%)	10 (8.26%)	
Concomitant use of IMM, *n* (%)				1.000
No	355 (58.58%)	284 (58.56%)	71 (58.68%)	
Yes	251 (41.42%)	201 (41.44%)	50 (41.32%)	
IFX products used by patients, *n* (%)				0.296
Product 1	595 (98.18%)	476 (98.14%)	119 (98.35%)	
Product 2	2 (0.33%)	1 (0.21%)	1 (0.83%)	
Product 3	3 (0.50%)	2 (0.41%)	1 (0.83%)	
≥2 products	6 (0.99%)	6 (1.24%)	0 (0.00%)	
Dosage (mg/kg), M (Q1, Q3)	5.71 (5.17, 6.19)	5.71 (5.17, 6.25)	5.71 (5.22, 6.12)	0.808
TLI (ug/mL), M (Q1, Q3)	4.59 (1.97, 11.35)	4.68 (2.02, 11.72)	4.00 (1.82, 10.20)	0.423
ESR (mm/h), M (Q1, Q3)	21.00 (11.00, 37.00)	22.00 (11.00, 38.00)	19.00 (9.00, 32.00)	0.158
CRP (mg/L), M (Q1, Q3)	4.41 (1.19, 16.43)	4.29 (1.16, 16.45)	5.00 (1.41, 15.86)	0.236
WBC ($\times 10^9$/L) , M (Q1, Q3)	6.07 (4.72, 7.59)	6.03 (4.71, 7.61)	6.11 (4.80, 7.41)	0.819
ANC ($\times 10^9$/L) , M (Q1, Q3)	3.81 (2.76, 5.23)	3.77 (2.70, 5.18)	3.83 (2.95, 5.27)	0.896

ALC (×10⁹/L) , M (Q1, Q3)	1.36 (1.07, 1.71)	1.36 (1.08, 1.71)	1.36 (1.02, 1.75)	0.800
RBC (×10¹²/L) , M (Q1, Q3)	4.64 (4.14, 5.06)	4.62 (4.11, 5.06)	4.75 (4.21, 5.06)	0.196
HB (g/L) , Mean ± SD	123.23 ± 20.42	122.82 ± 20.46	124.88 ± 20.27	0.319
PLT (×10⁹/L) , M (Q1, Q3)	289.00 (233.00, 350.98)	290.00 (232.60, 361.00)	286.00 (234.00, 323.80)	0.191
AMC (×10⁹/L) , M (Q1, Q3)	0.50 (0.37, 0.65)	0.50 (0.38, 0.66)	0.49 (0.36, 0.63)	0.441
HCT, M (Q1, Q3)	0.39 (0.35, 0.42)	0.39 (0.35, 0.42)	0.39 (0.36, 0.42)	0.293
ALT (U/L), M (Q1, Q3)	14.54 (9.86, 23.05)	14.27 (9.65, 23.09)	15.99 (10.47, 22.82)	0.324
AST (U/L), M (Q1, Q3)	17.75 (14.05, 23.92)	17.55 (14.03, 23.38)	18.37 (14.18, 24.97)	0.510
GGT (U/L), M (Q1, Q3)	18.92 (14.01, 28.96)	18.72 (14.02, 28.82)	19.17 (13.88, 29.47)	0.863
ALP (U/L), M (Q1, Q3)	79.78 (66.34, 95.25)	79.84 (66.35, 95.47)	79.76 (65.94, 92.29)	0.702
TBIL (umol/L), M (Q1, Q3)	10.12 (7.77, 13.80)	10.23 (7.78, 13.90)	9.82 (7.72, 13.09)	0.348
DBIL (umol/L), M (Q1, Q3)	2.12 (1.58, 2.90)	2.14 (1.60, 2.95)	2.01 (1.50, 2.64)	0.164
IBIL (umol/L), M (Q1, Q3)	7.92 (6.14, 10.87)	7.96 (6.13, 10.98)	7.89 (6.16, 10.31)	0.464
TC (mmol/L), M (Q1, Q3)	3.59 (3.11, 4.20)	3.57 (3.11, 4.20)	3.63 (3.11, 4.21)	0.636
K (mmol/L), Mean ± SD	3.99 ± 0.32	3.99 ± 0.31	3.97 ± 0.35	0.414
Na (mmol/L), Mean ± SD	139.84 ± 2.10	139.84 ± 2.16	139.80 ± 1.86	0.826
Ca (mmol/L), M (Q1, Q3)	2.29 (2.20, 2.38)	2.29 (2.21, 2.38)	2.28 (2.16, 2.39)	0.206
P (mmol/L), Mean ± SD	1.26 ± 0.22	1.26 ± 0.22	1.25 ± 0.19	0.565
UA (umol/L), Mean ± SD	355.64 ± 106.08	357.41 ± 108.64	348.56 ± 95.25	0.376
Cr (umol/L), M (Q1, Q3)	71.37 (59.65, 79.98)	70.99 (59.95, 80.04)	72.59 (58.05, 78.17)	0.806
ALB (g/L) , Mean ± SD	39.76 ± 5.20	39.83 ± 5.10	39.47 ± 5.58	0.510

BMI, body mass index; IFX, infliximab; CDAI, the Crohn's disease activity index; L1, ileal; L2, colonic; L3, ileocolonic location of disease; L4, upper gastrointestinal; B1, inflammatory disease; B2, stricturing disease, B3, penetrating disease; EIM, extraintestinal manifestations; IMM, immunosuppressants; TLI, serum trough levels of IFX; ESR, erythrocyte sedimentation rate; CRP, C-reactive protein; WBC, white blood cell count; ANC, absolute neutrophil count; ALC, absolute lymphocyte count; RBC, red blood cell count; HB, hemoglobin; PLT, platelet count; AMC, absolute monocyte count; HCT, hematocrit; ALT, alanine aminotransferase; AST, aspartate aminotransferase; GGT, gamma-glutamyl transferase; ALP, alkaline phosphatase; TBIL, total bilirubin; DBIL, direct bilirubin; IBIL, indirect bilirubin; TC, total cholesterol; K, potassium; Na, sodium; Ca, calcium; P, phosphorus; UA, uric acid; Cr, creatinine; ALB, albumin.

Table S2. Comparison of other clinical characteristics between ADA-positive and ADA-negative patients during IFX induction therapy in the training set.

Variables	Total (*n*=485)	ADA negative (*n*=369)	ADA positive (*n*=116)	*p* value
Height (cm), M (Q1, Q3)	169.00 (163.00, 173.00)	169.00 (164.00, 173.00)	170.00 (162.00, 172.00)	0.630
Weight (kg) , M (Q1, Q3)	54.00 (48.00, 61.00)	55.00 (50.00, 61.00)	51.50 (46.75, 60.00)	0.012
IFX products used by patients, *n* (%)				0.410
Product 1	476 (98.14%)	360 (97.56%)	116 (100.00%)	
Product 2	1 (0.21%)	1 (0.27%)	0 (0.00%)	
Product 3	2 (0.41%)	2 (0.54%)	0 (0.00%)	
≥2 products	6 (1.24%)	6 (1.63%)	0 (0.00%)	
CRP (mg/L) , M (Q1, Q3)	4.29 (1.16, 16.45)	3.63 (0.99, 15.19)	5.72 (1.55, 19.74)	0.102
WBC (×109/L) , M (Q1, Q3)	6.03 (4.71, 7.61)	6.03 (4.71, 7.65)	6.05 (4.70, 7.43)	0.701
ANC (×109/L) , M (Q1, Q3)	3.77 (2.70, 5.18)	3.81 (2.70, 5.23)	3.68 (2.75, 5.06)	0.874
ALC (×109/L) , M (Q1, Q3)	1.36 (1.08, 1.71)	1.38 (1.08, 1.72)	1.34 (1.04, 1.60)	0.294
RBC (×1012/L) , M (Q1, Q3)	4.62 (4.11, 5.06)	4.62 (4.15, 5.06)	4.59 (4.05, 5.09)	0.237
HB (g/L) , Mean ± SD	122.82 ± 20.46	123.87 ± 19.97	119.47 ± 21.69	0.054
PLT (×109/L) , M (Q1, Q3)	290.00 (232.60, 361.00)	284.00 (232.00, 351.30)	312.50 (244.50, 374.75)	0.122
AMC (×109/L) , M (Q1, Q3)	0.50 (0.38, 0.66)	0.50 (0.37, 0.66)	0.52 (0.38, 0.66)	0.583
HCT, M (Q1, Q3)	0.39 (0.35, 0.42)	0.39 (0.35, 0.42)	0.38 (0.33, 0.41)	0.066
ALT (U/L) , M (Q1, Q3)	14.27 (9.65, 23.09)	14.18 (9.58, 21.73)	14.68 (10.44, 26.81)	0.167
AST (U/L) , M (Q1, Q3)	17.55 (14.03, 23.38)	17.45 (14.13, 22.72)	17.86 (13.96, 25.94)	0.480
GGT (U/L) , M (Q1, Q3)	18.72 (14.02, 28.82)	17.96 (14.01, 28.31)	22.41 (14.73, 32.59)	0.109
ALP (U/L) , M (Q1, Q3)	79.84 (66.35, 95.47)	80.20 (66.53, 97.00)	79.12 (65.82, 90.85)	0.318
TBIL (umol/L) , M (Q1, Q3)	10.23 (7.78, 13.90)	10.51 (7.80, 13.99)	9.75 (7.64, 13.76)	0.295
DBIL (umol/L) , M (Q1, Q3)	2.14 (1.60, 2.95)	2.12 (1.63, 3.00)	2.18 (1.54, 2.87)	0.649
IBIL (umol/L) , M (Q1, Q3)	7.96 (6.13, 10.98)	8.16 (6.10, 11.08)	7.50 (6.18, 10.85)	0.260
TC (mmol/L) , M (Q1, Q3)	3.57 (3.11, 4.20)	3.50 (3.12, 4.18)	3.70 (3.08, 4.22)	0.469

K (mmol/L) , Mean ± SD	3.99 ± 0.31	3.99 ± 0.31	4.01 ± 0.31	0.621
Na (mmol/L) , Mean ± SD	139.84 ± 2.16	139.94 ± 2.01	139.54 ± 2.58	0.129
P (mmol/L) , Mean ± SD	1.26 ± 0.22	1.26 ± 0.23	1.27 ± 0.22	0.760
UA (umol/L) , Mean±SD	357.41±108.64	355.59±107.34	363.19±112.94	0.523
Cr (umol/L) , M (Q1, Q3)	70.99 (59.95, 80.04)	72.29 (61.96, 80.08)	67.62 (56.63, 78.52)	0.097

BMI, body mass index; IFX, infliximab; ADA, anti-drug antibodies; ESR, erythrocyte sedimentation rate; CRP, C-reactive protein; WBC, white blood cell count; ANC, absolute neutrophil count; ALC, absolute lymphocyte count; RBC, red blood cell count; HB, hemoglobin; PLT, platelet count; AMC, absolute monocyte count; HCT, hematocrit; ALT, alanine aminotransferase; AST, aspartate aminotransferase; GGT, gamma-glutamyl transferase; ALP, alkaline phosphatase; TBIL, total bilirubin; DBIL, direct bilirubin; IBIL, indirect bilirubin; TC, total cholesterol; K, potassium; Na, sodium; P, phosphorus; UA, uric acid; Cr, creatinine.

Table S3. Hyperparameter configurations for machine learning models

ML models	Hyperparameter settings
LR	family = “binomial”
RF	mtry = 2, ntree= 100, importance=TRUE, tuneLength=10
KNN	kernel= “triangular”, k=27
CART	cp = 0.3, method= “class”
XGBoost	max_depth = 3, eta = 0.1, nrounds = 50
LightGBM	learning_rate = 0.05, n_estimators = 200, num_leaves = 63, max_depth = 7
SVM	kernel= “radial”, cost = 1000, gamma = 0.001, probability=TRUE

LR, logistic regression; RF, random forest; KNN, k-nearest neighbors; CART, classification and regression tree; XGBoost, extreme gradient boosting; LightGBM, light gradient boosting machine; SVM, support vector machine.

Table S4. Model performance in predicting ADA positivity during IFX induction therapy in the training and validation sets

Model	AUC (95% CI)	Accuracy (95% CI)	Sensitivity	Specificity	PPV	NPV	F1	Brier
Training set								
LR	0.836 (0.794, 0.878)	0.827 (0.790, 0.859)	0.500	0.930	0.690	0.855	0.580	0.127
RF	0.987 (0.980, 0.994)	0.934 (0.908, 0.954)	0.733	0.997	0.988	0.922	0.842	0.048
KNN	0.905 (0.877, 0.932)	0.845 (0.810, 0.876)	0.526	0.946	0.753	0.864	0.619	0.127
CART	0.762 (0.716, 0.809)	0.872 (0.839, 0.900)	0.552	0.973	0.865	0.873	0.674	0.112
XGBoost	0.935 (0.910, 0.961)	0.903 (0.873, 0.928)	0.672	0.976	0.897	0.905	0.768	0.077
LightGBM	0.919 (0.891, 0.948)	0.858 (0.823, 0.888)	0.853	0.859	0.656	0.949	0.742	0.109
SVM	0.890 (0.849, 0.932)	0.889 (0.857, 0.915)	0.586	0.984	0.919	0.883	0.565	0.143
Validation set								
LR	0.875 (0.791, 0.960)	0.851 (0.775, 0.909)	0.586	0.935	0.739	0.878	0.654	0.116
RF	0.865 (0.777, 0.954)	0.843 (0.766, 0.903)	0.517	0.946	0.750	0.861	0.612	0.117
KNN	0.881 (0.804, 0.958)	0.860 (0.785, 0.916)	0.586	0.946	0.773	0.879	0.667	0.116
CART	0.697 (0.602, 0.792)	0.826 (0.747, 0.889)	0.448	0.946	0.722	0.845	0.553	0.145
XGBoost	0.899 (0.841, 0.957)	0.851 (0.775, 0.909)	0.551	0.946	0.762	0.946	0.640	0.102
LightGBM	0.888 (0.824, 0.953)	0.826 (0.747, 0.889)	0.759	0.848	0.611	0.918	0.677	0.123
SVM	0.735 (0.602, 0.868)	0.835 (0.756, 0.896)	0.448	0.957	0.765	0.846	0.565	0.143

ADA, anti-drug antibodies; IFX, infliximab; LR, logistic regression; RF, random forest; KNN, k-nearest neighbors; CART, classification and regression tree; XGBoost, extreme gradient boosting; LightGBM, light gradient boosting machine; SVM, support vector machine; PPV, positive predictive value; NPV, negative predictive value.

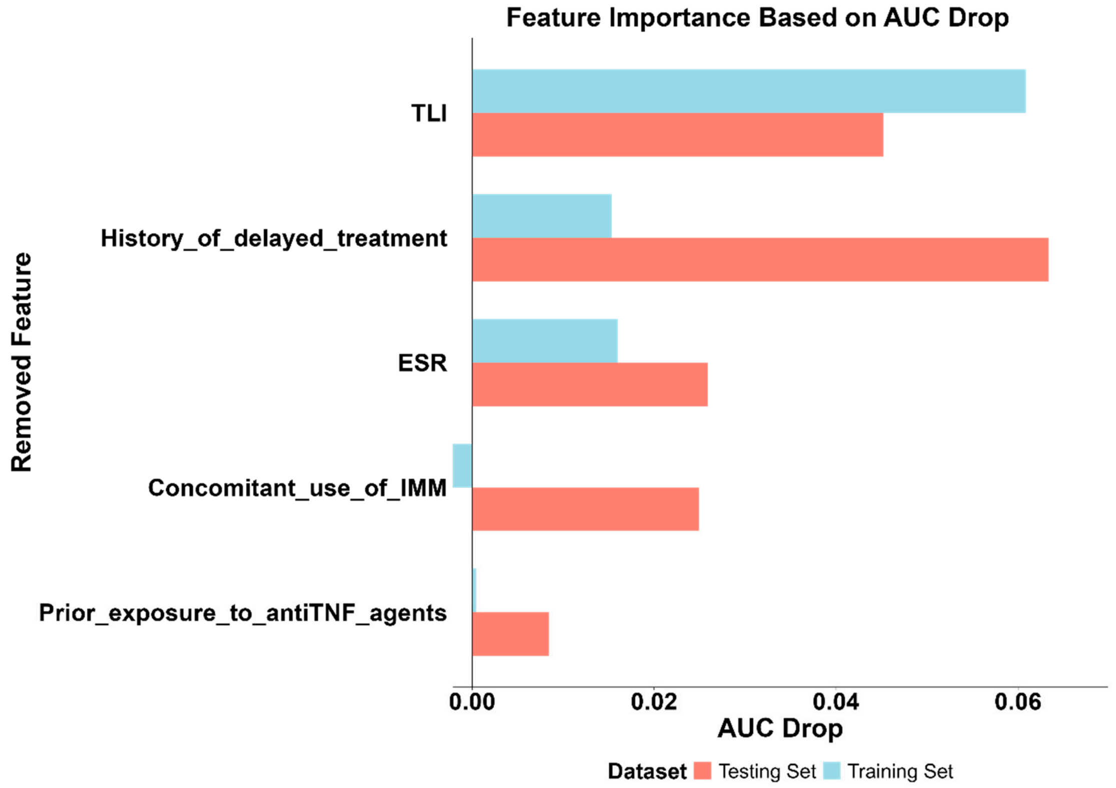

Figure S2. Key predictors of the XGBoost model identified through ablation study

Table S5. Ablation study of the XGBoost model: performance evaluation on training and testing sets

Removed feature	Dataset	AUC	95% CI	AUC drop	Mean AUC drop
Without TLI	Training	0.877	(0.827, 0.927)	0.061	0.055
Without TLI	Testing	0.854	(0.804, 0.904)	0.045	
Without History of delayed treatment	Training	0.922	(0.872, 0.972)	0.015	0.030
Without History of delayed treatment	Testing	0.836	(0.786, 0.886)	0.063	
Without ESR	Training	0.922	(0.872, 0.972)	0.016	0.018
Without ESR	Testing	0.873	(0.823, 0.923)	0.026	
Without Concomitant use of IMM	Training	0.940	(0.890, 0.990)	-0.002	0.013
Without Concomitant use of IMM	Testing	0.874	(0.824, 0.924)	0.025	
Without Prior exposure to anti-TNF agents	Training	0.937	(0.887, 0.987)	0.000	0.014
Without Prior exposure to anti-TNF agents	Testing	0.891	(0.841, 0.941)	0.008	

TLI, serum trough levels of infliximab; ESR, erythrocyte sedimentation rate; IMM, immunosuppressants.

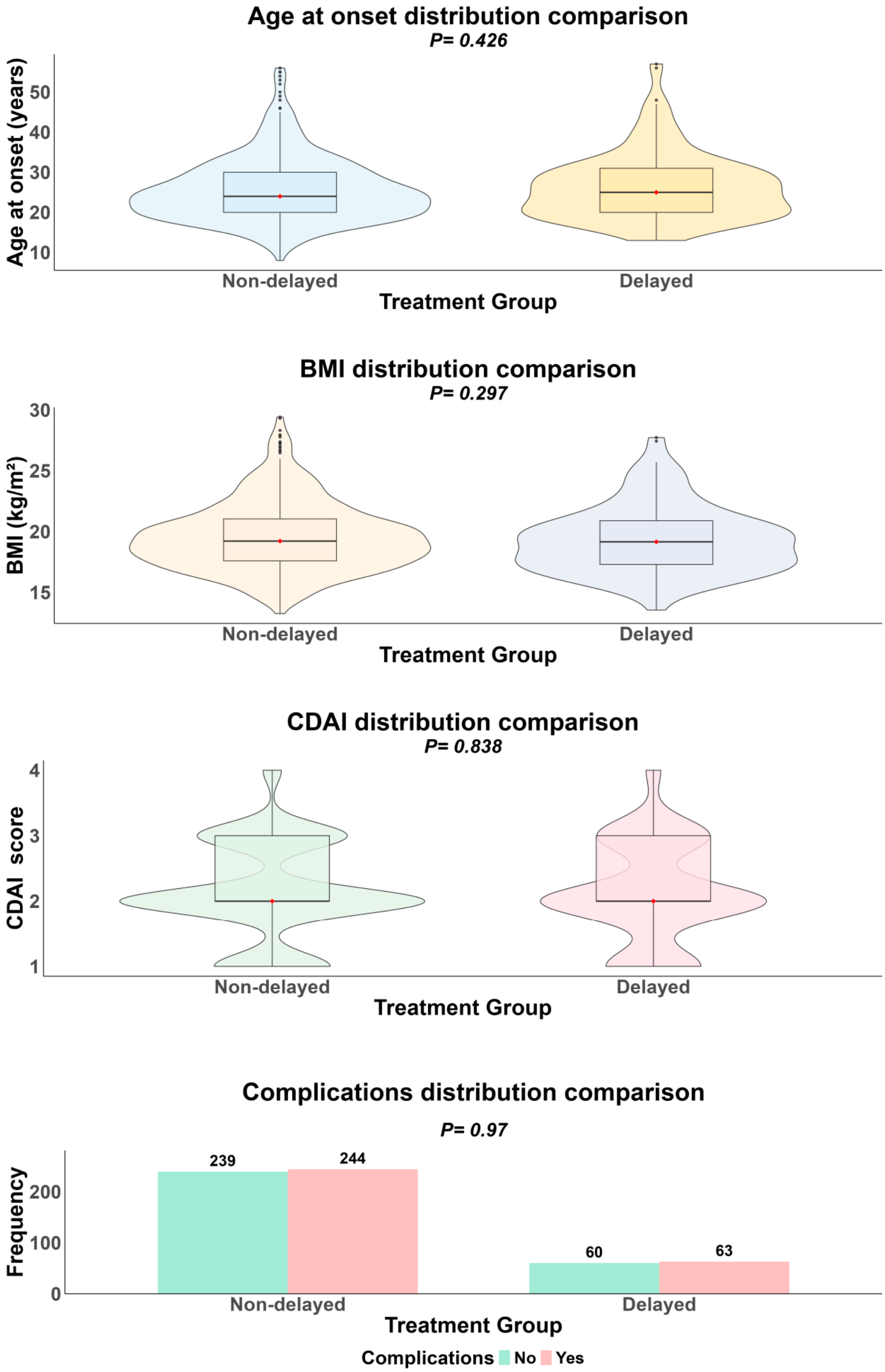

Figure S3. Comparison of CDAI score, age at onset, BMI, and complication frequencies between the delayed-treatment group and the non-delayed-treatment group.

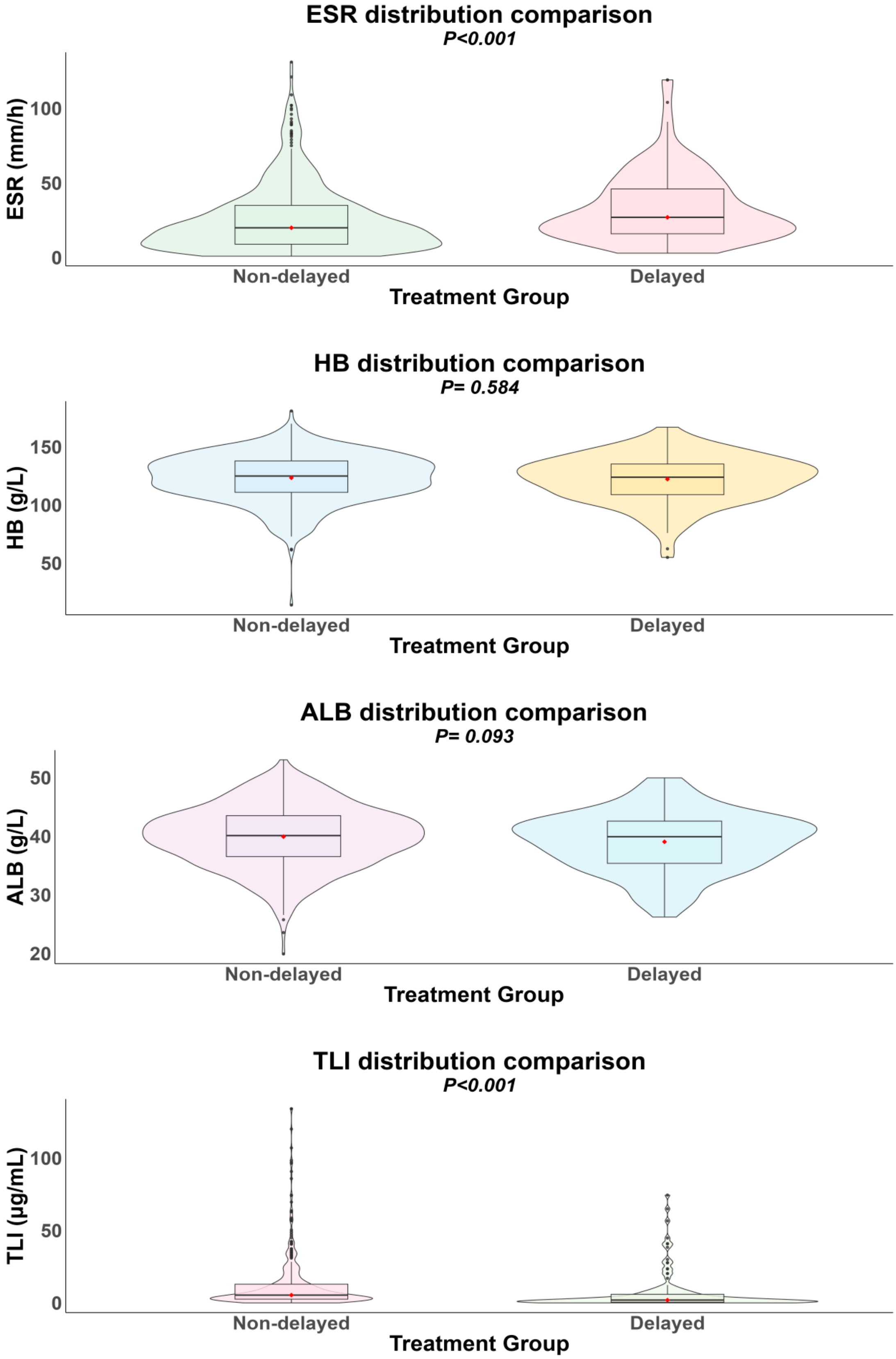

Figure S4. Comparisons of erythrocyte sedimentation rate (ESR), hemoglobin (HB), albumin (ALB), and serum trough levels of infliximab (TLI) between the delayed-treatment group and the non-delayed-treatment group.

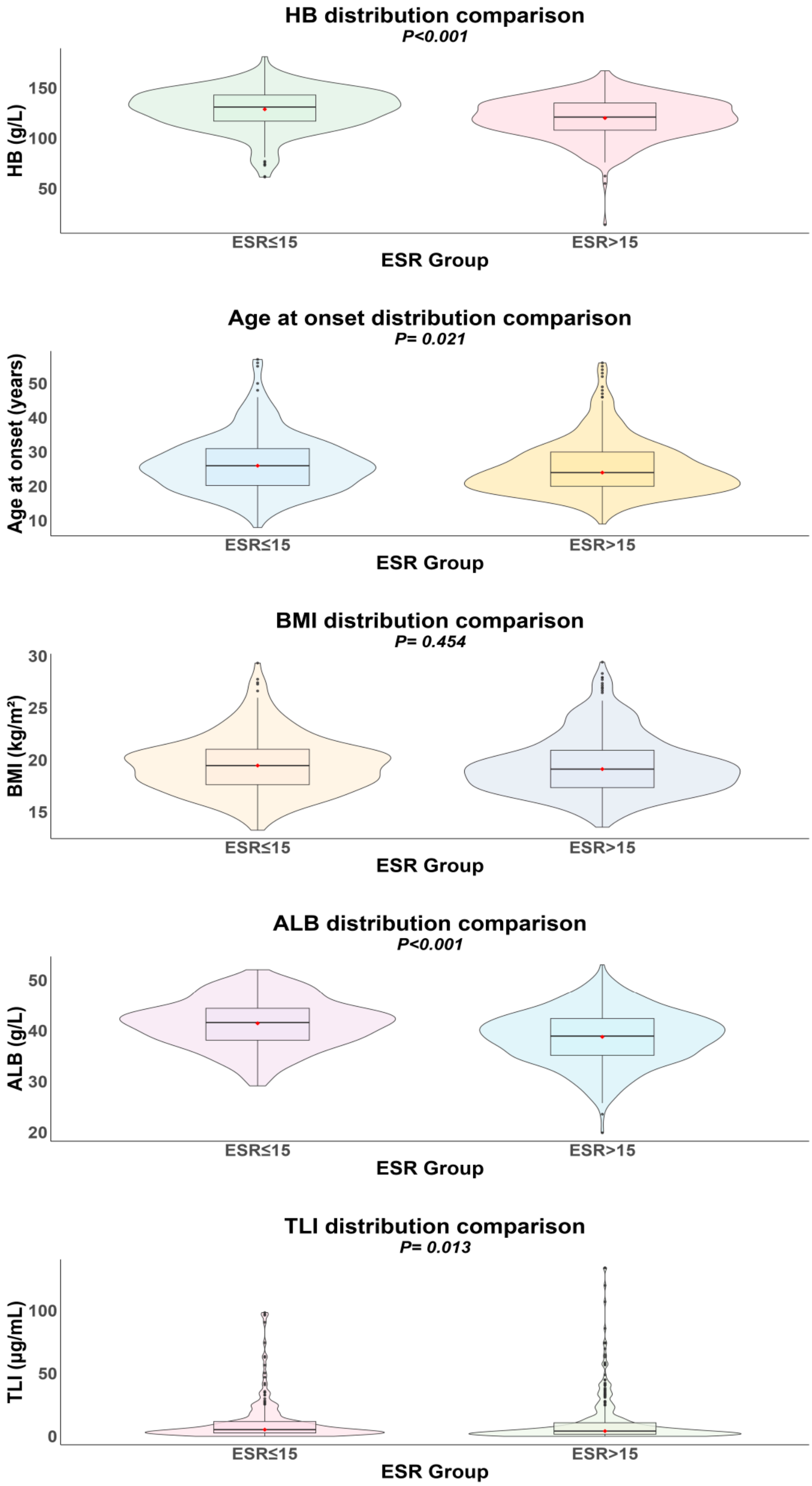

Figure S5. Comparison of hemoglobin (HB), age at onset, BMI, albumin (ALB), and serum trough levels of infliximab (TLI) stratified by erythrocyte sedimentation rate (ESR) level (>15 mm/h vs. ≤15 mm/h).
